# How Can We Overcome the Strength–Ductility Tradeoff in Light Alloys and Related Composites?

**DOI:** 10.3390/ma16030934

**Published:** 2023-01-18

**Authors:** Tong Gao, Xiangfa Liu

**Affiliations:** Key Laboratory for Liquid–Solid Structural Evolution and Processing of Materials, Ministry of Education, Shandong University, 17923 Jingshi Road, Jinan 250061, China

## Introduction

In recent decades, the design and development of light alloys and related composites to achieve a good combination of strength and ductility have attracted huge attention. The most commonly covered topics include the selection of the matrix, the content of the reinforcements, the development of synthesis methods, strengthening mechanisms, etc. However, with an increase in strength, ductility usually decreases sharply and vice versa, i.e., there a well-documented strength–ductility tradeoff [1]. This issue has become an impenetrable dilemma for researchers.

Using a heterogeneous rather than a homogeneous structure seems to be a useful method of solving the above issue. Y.T. Zhu et al. defined the concept of ‘heterostructured materials’ [2]. Heterostructured materials contain a series of hetero-zones, and interactions in these hetero-zones can produce a synergistic effect where the integrated property exceeds the prediction by the rule-of-mixtures. These materials usually have heterogeneous lamella structures [3], gradient structures [4], laminate structures [5], dual/multi–phase structures [6], harmonic (core–shell) structures [7], multimodal structures [8], etc. They possess superior combinations of strength and ductility, which can be attributed to hetero-deformation-induced strengthening and strain hardening.

Another useful approach focuses on the control of reinforcements in materials, and the most representative work can be found in the work of Z.P. Lu’s research group [9,10]. It is known that the commonly occurring precipitates in light alloys and related composites contribute to an increase in strength while unavoidably creating large coherency strains, which in turn may promote crack initiation under load. Z.P. Lu et al. [9] utilized highly dispersed and fully coherent precipitates (with minimal lattice misfit with the matrix) to strengthen steel materials while not sacrificing their ductility. In another work [10], they achieved ordered oxygen complexes in high-entropy alloys (HEAs). In contrast to traditional interstitial strengthening, such ordered interstitial complexes lead to unprecedented enhancements in both strength and ductility.

The above two concepts, by controlling either the heterostructures of materials or the form of reinforcement, shed new light on the design of advanced light alloys and related composites. On the one hand, successfully applying these concepts to material synthesis is a challenge. On the other hand, new concepts that can help us to overcome the strength–ductility tradeoff issue are always interesting for researchers.

Although light alloys and related composites have been investigated for decades, there are still challenges to overcome. All investigations into the design, preparation, characterization, modeling, and testing of metallic alloys or composites are welcome in the current Special Issue entitled “Advances in Light Alloys and Related Composites”.

## Data Availability

Not applicable.
